# Supplementary figures and images for: The mammalian tRNA ligase complex mediates splicing of XBP1 mRNA and controls antibody secretion in plasma cells
Source: EMBO J. 2014 Nov 6;33(24):2922–36. doi: 10.15252/embj.201490332 (PMC4282640; doi:10.15252/embj.201490332)

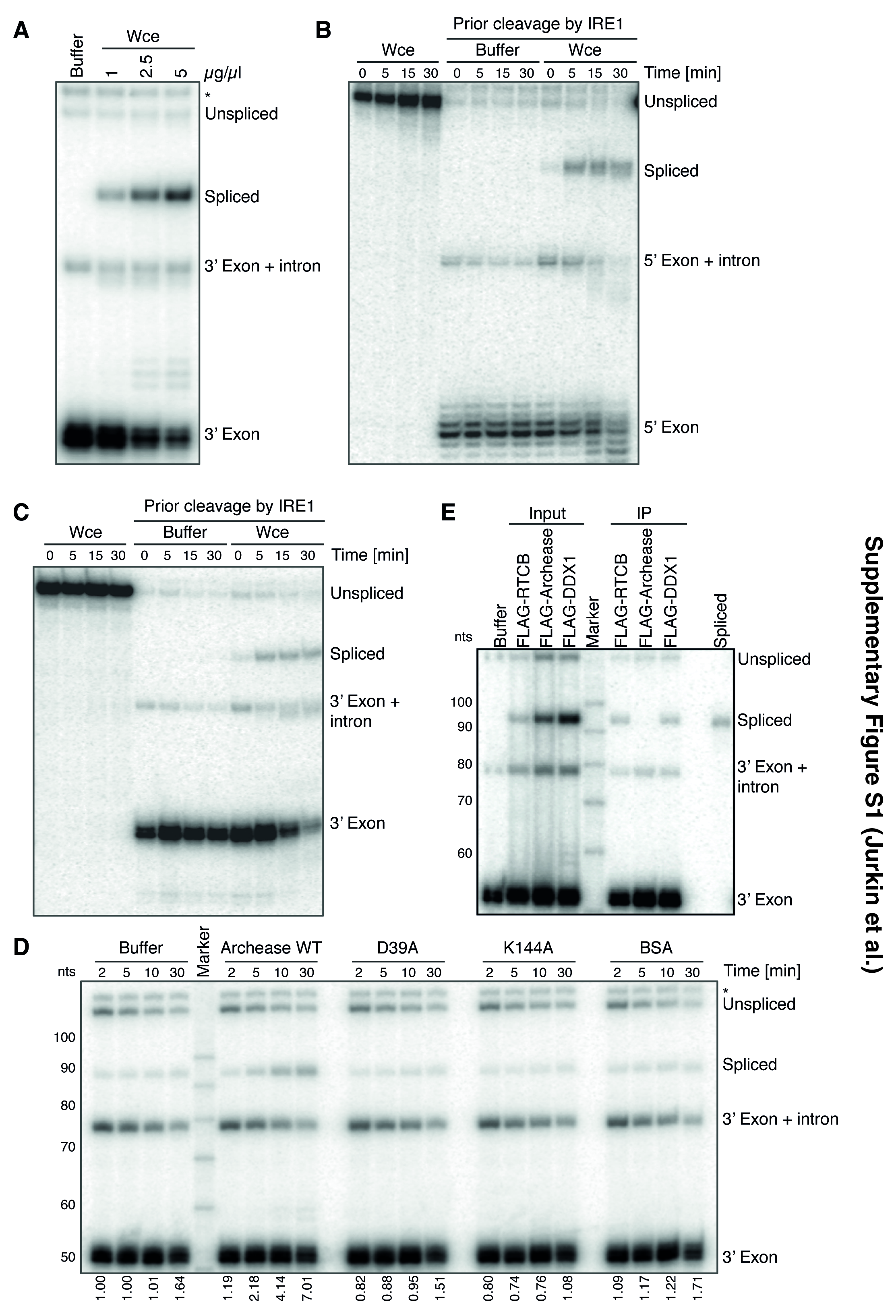

Supplement: Supplementary file 1 — Supplementary Figure S1 [file embj0033-2922-sd1.tif]

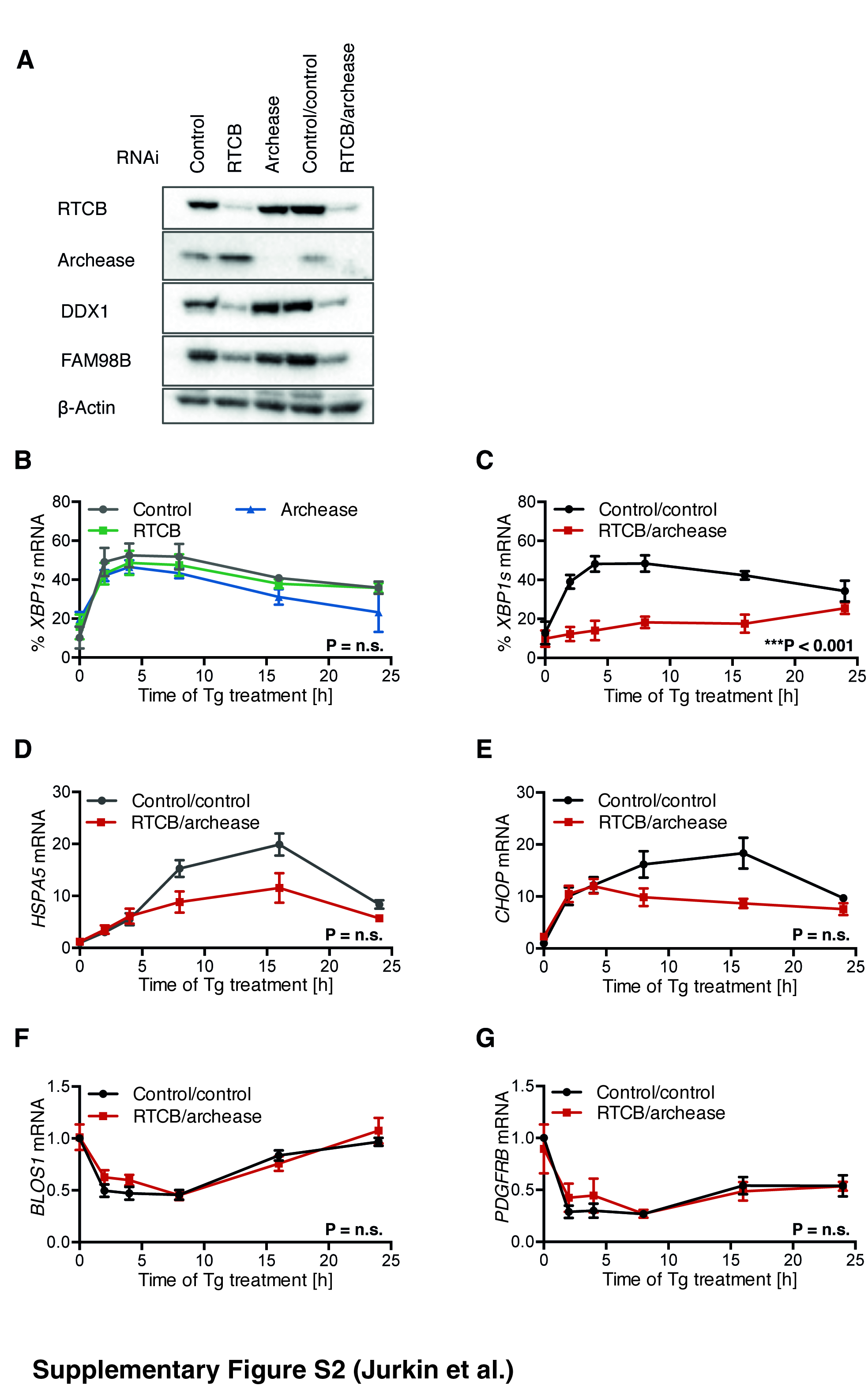

Supplement: Supplementary file 2 — Supplementary Figure S2 [file embj0033-2922-sd2.tif]

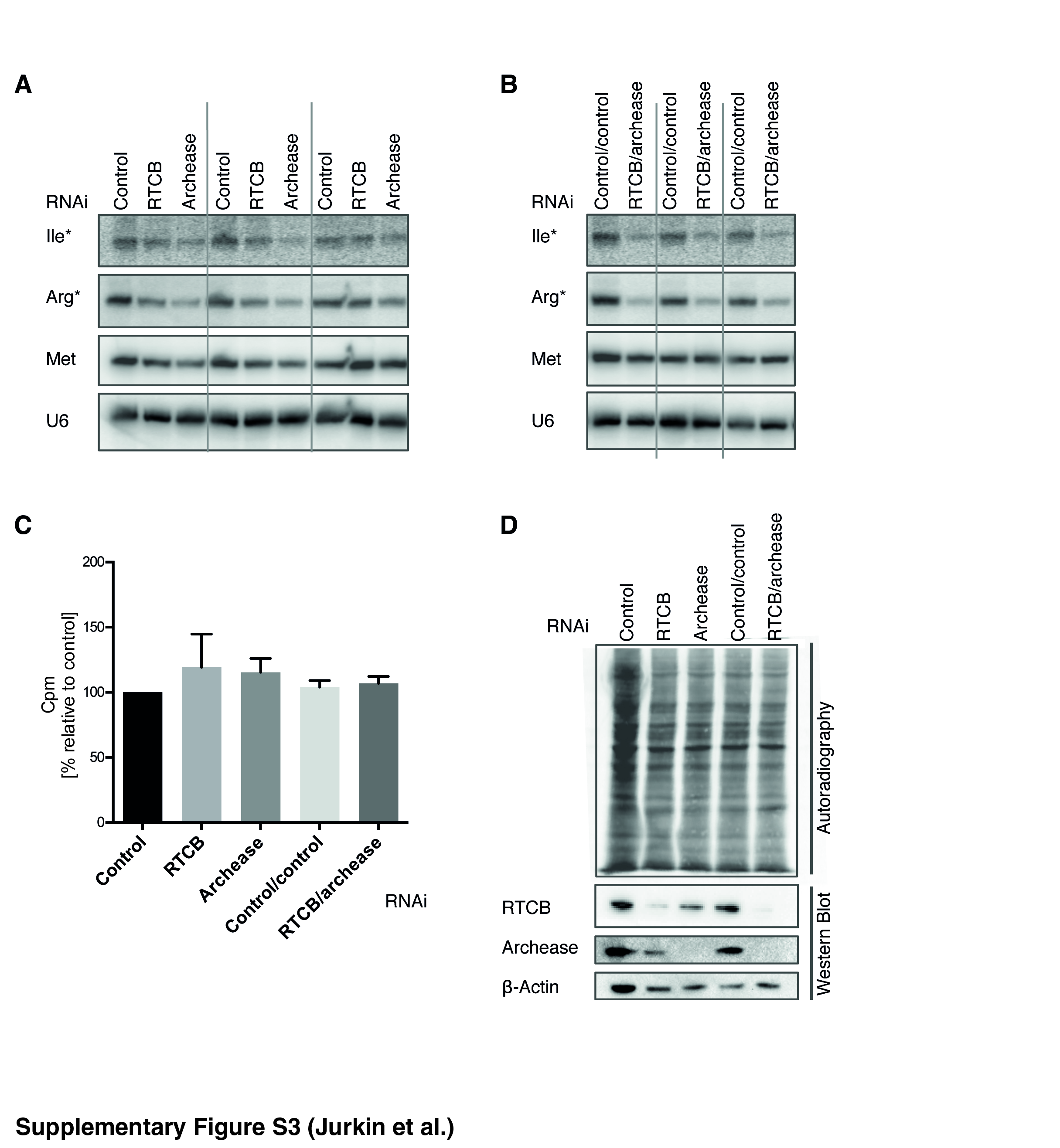

Supplement: Supplementary file 3 — Supplementary Figure S3 [file embj0033-2922-sd3.tif]

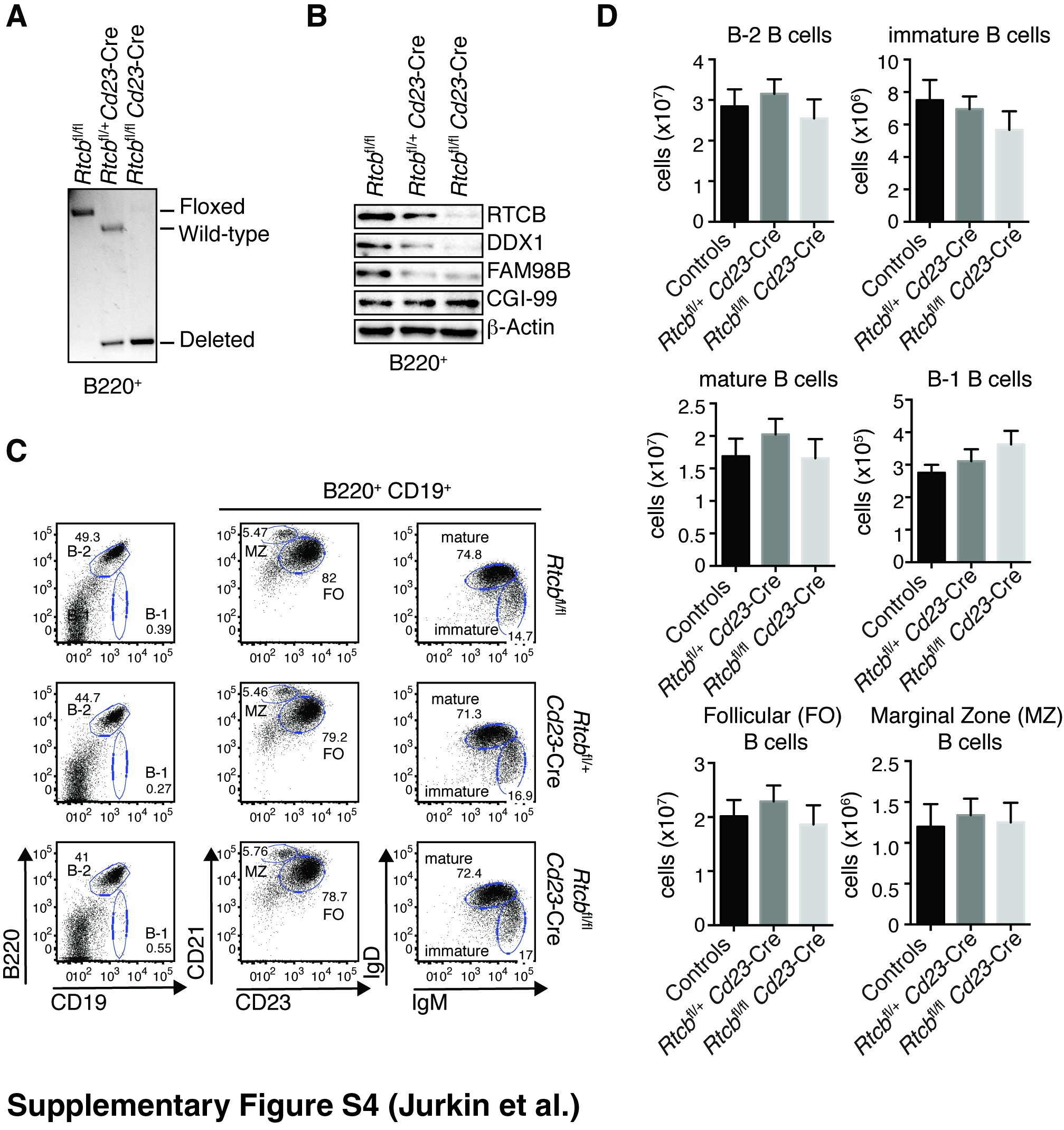

Supplement: Supplementary file 4 — Supplementary Figure S4 [file embj0033-2922-sd4.tif]

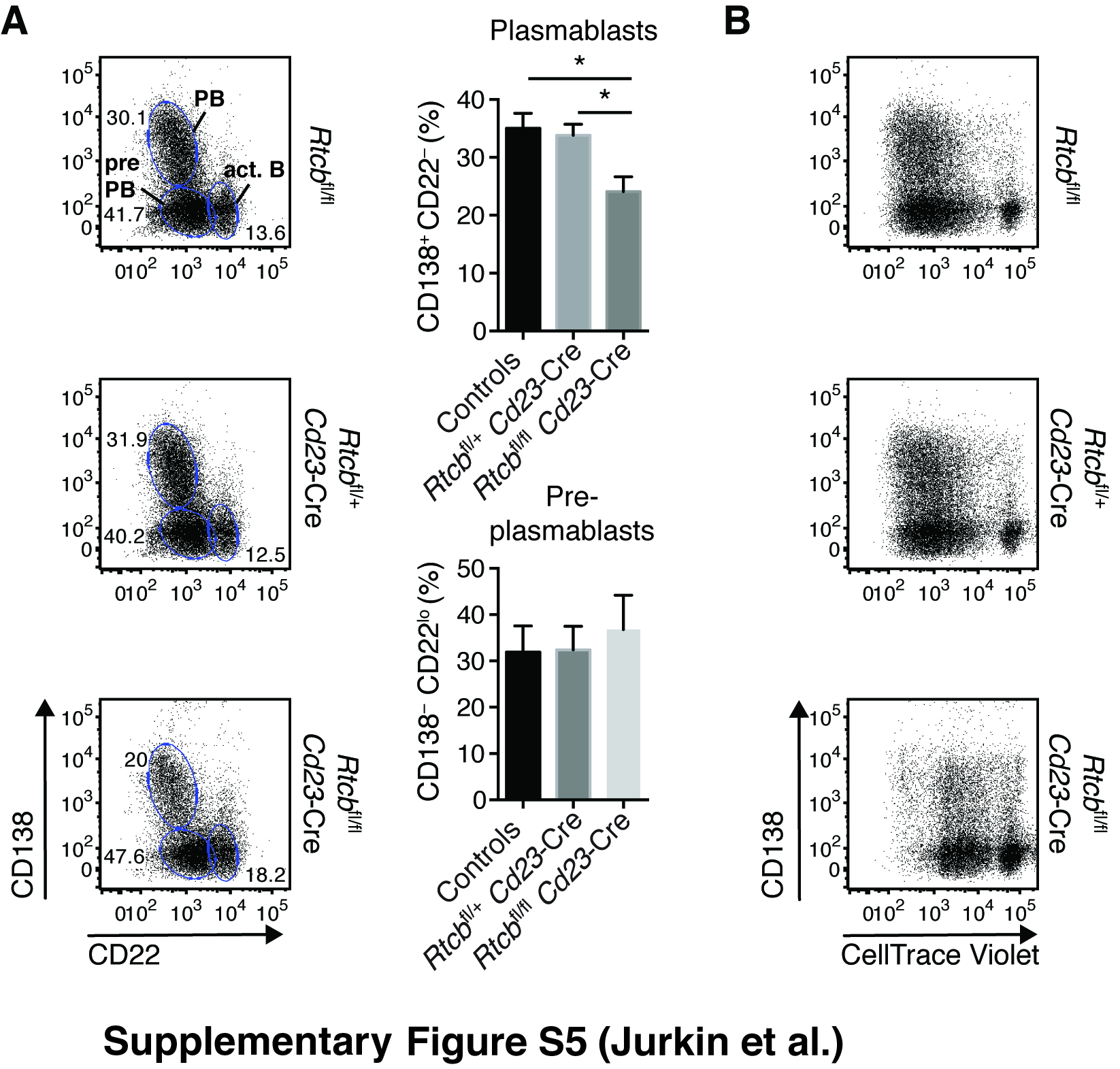

Supplement: Supplementary file 5 — Supplementary Figure S5 [file embj0033-2922-sd5.tif]

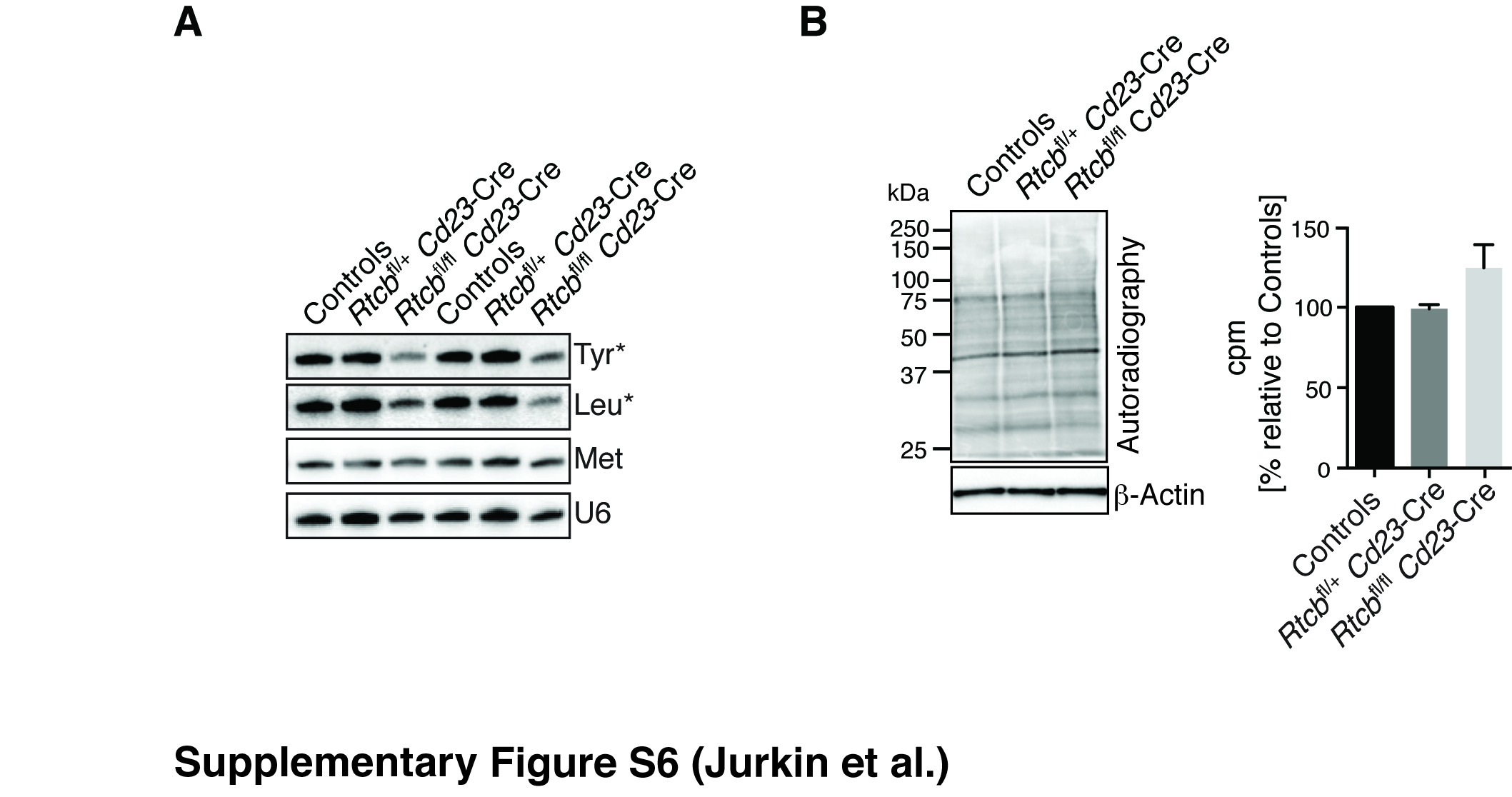

Supplement: Supplementary file 6 — Supplementary Figure S6 [file embj0033-2922-sd6.tif]
