# Supplementary material for: The mammalian tRNA ligase complex mediates splicing of XBP1 mRNA and controls antibody secretion in plasma cells
Source: EMBO J. 2014 Nov 6;33(24):2922–36. doi: 10.15252/embj.201490332 (PMC4282640; doi:10.15252/embj.201490332)
Supplement: Supplementary file 7 — Legends for Supplementary Figures [file embj0033-2922-sd7.pdf]

Supplementary Figure Legends

Figure S1 | *XBP1* mRNA ligation assay

A An internally labeled fragment of human *XBP1* mRNA including the intron was pre-cleaved with recombinant IRE1 endonuclease and afterwards supplemented with buffer or different concentrations of HeLa whole cell extracts for 15 min to monitor *XBP1* mRNA ligation activity. An unspecific band is marked with an asterix.

B,C, *In vitro XBP1* mRNA ligation assay using 5' end- (**B**) or 3' end- labeled (**C**) *XBP1* mRNA fragment as substrate.

D, A 3' end-labeled *XBP1* mRNA fragment was pre-cleaved with recombinant IRE1 and incubated with lysates of cells overexpressing wild-type or mutant forms of archease. Amounts of spliced *XBP1* mRNA fragment formed during the reaction were quantified and normalized relative to the sample supplemented with buffer for 2 min. An unspecific band is marked with an asterix.

E, *XBP1* mRNA ligation assay using a 3' end-labeled *XBP1* mRNA fragment incubated for 15 min with HeLa whole cell extracts (Input) or immunoprecipitates (IP) of RTCB, archease and DDX1.

Figure S2 | Protein and mRNA levels after RTCB and/or archease depletion

A, After six days of Dox treatment, Tet-ON HeLa cells expressing shRNAs against RTCB, archease or both and control cell lines expressing one or two copies of the control shRNA were lysed and RTCB, archease, DDX1, and FAM98B expression levels were analyzed by Western Blot (n=3).

B, Quantification of RT-PCR experiments performed after expression of shRNAs targeting control, RTCB or archease in HeLa cells (n=3, mean expression levels and SEM are displayed). *XBP1* mRNA splicing was induced by treatment with 300 nM Tg for the indicated time periods. Amounts of *XBP1s* and *XBP1u* mRNA were quantified by densitometry after semiquantitative PCR.

C, Quantification of RT-PCR experiments performed upon shRNA-mediated, simultaneous depletion of RTCB and archease in HeLa cells (n=5, mean expression levels and SEM are displayed).

D-G, Tet-ON HeLa cell lines coding for shRNAs targeting RTCB and archease or the control cell line expressing two copies of the control shRNA were treated with Dox (1 µg/ml, six days) and Tg (300 nM, 24 h time course). Relative mRNA levels of *HSPA5* and *CHOP* as well as of the RIDD target-genes *BLOS1* and *PDGFRB* were analyzed by RT-qPCR (n=5, mean expression levels and SEM are displayed). Expression levels were normalized to *ACTB* mRNA levels and to the untreated control sample. Two-way ANOVA was used to analyze statistical significance of differences in mRNA levels between control and RTCB/archease depleted cells (*P < 0.05, **P < 0.01, ***P < 0.001, ****P < 0.0001).

Figure S3 | Depletion of RTCB and archease reduces mature tRNA levels of splicing-dependent tRNAs.

A,B, After six days of Dox treatment, RNA was isolated from Tet-ON HeLa cells expressing shRNAs against RTCB, archease or both and from control cell lines expressing one or two copies of the control shRNA. Northern Blot analysis of total RNA was performed using DNA probes complementary to the indicated mature tRNAs. Intron-containing tRNAs are marked with an asterix (n=3).

C, After six days of Dox treatment, Tet-ON HeLa cells expressing shRNAs against RTCB, archease or both and control cell lines expressing one or two copies of the control shRNA were incubated with ^{35}S labeled methionine and cysteine for one hour and subsequently lysed. Protein translation efficiency was analyzed by scintillation counting (cpm, counts per minute) of the cell lysates and normalized to the protein concentration as evaluated by BCA assay and to the control sample obtained from Tet-ON HeLa cells expressing only one copy of the control shRNA (n=2, mean and SD are displayed).

D, Lysates obtained after metabolic labeling were resolved by SDS-PAGE and analyzed by autoradiography. β-actin was used as a loading control. Expression levels of RTCB and archease were likewise visualized by Western blot (n=2).

Figure S4 | Initial characterization of *Rtcb*$^{fl/fl}$ *Cd23*-Cre mice

A,B, B220^{+} cells were enriched by magnetic cell sorting from spleens of control (*Rtcb*$^{fl/fl}$ or *Rtcb*$^{fl/+}$), heterozygous (*Rtcb*$^{fl/+}$ *Cd23*-Cre) or homozygous (*Rtcb*$^{fl/fl}$ *Cd23*-Cre) mice and analyzed by PCR genotyping **(A)** or Western blot analysis **(B)** for deletion efficiency (n>3).

C,D, Spleens of the indicated genotypes were analyzed by flow cytometry for the presence of different B cell populations. Representative dot plots are shown in **C**. Bar diagrams in **D** show total cell numbers (n>6 for each genotype, mean and SEM are displayed). Populations were defined as B220$^{low/–}$ CD19^{+} (B-1 B cells), B220^{+} CD19^{+} (B-2 B cells), B220^{+} CD19^{+} CD23^{low} CD21^{high} (marginal zone B cells), B220^{+} CD19^{+} CD23^{high} CD21^{Int} (follicular B cells), B220^{+} CD19^{+} IgM^{high} IgD^{low} (immature B cells), B220^{+} CD19^{+} IgM^{low} IgD^{high} (mature B cells).

Figure S5 | *Rtcb*$^{fl/fl}$ *Cd*23-Cre B cells show reduced proliferation rates

A,B, Splenic B220^{+} B cells isolated from control (*Rtcb*$^{fl/fl}$ or *Rtcb*$^{fl/+}$), *Rtcb*$^{fl/+}$ *Cd23*-Cre or *Rtcb*$^{fl/fl}$ *Cd23*-Cre mice were labeled with the CellTrace Violet reagent, stimulated for 4 days with LPS and analyzed by flow cytometry (PB: plasmablast; pre-PB: pre-plasmablast; actB: activated B cell). The percentages of CD138^{+} CD22$^{–}$ plasmablasts or CD138$^{–}$ CD22^{low} pre-plasmablasts are shown in **A.** Representative dot plots (left panel) and bar diagrams (right panel) representing mean percentages and SEM (n=4) are displayed. Flow cytometric analysis of CellTrace Violet dilution of unfractionated cells stimulated for 4 days with LPS is presented in **B** (n=4). An unpaired student's t-test was used to analyze the statistical significance (*$P < 0.05$).

Figure S6 | *Rtcb*$^{fl/fl}$ *Cd23*-Cre B cells have reduced mature tRNA levels of splicing-dependent tRNAs, but do not display defects in global protein synthesis

A, Northern Blot analysis of day-4 LPS-stimulated B220^{+} B cells with probes against the indicated mature tRNAs. Intron-containing tRNAs are marked with an asterisk (n=4).

B, B cells stimulated with LPS for 4 days were incubated with ^{35}S-labeled methionine and cysteine for 1 h and subsequently lysed. The protein translation efficiency was analyzed by SDS-PAGE followed by autoradiography. Bar diagrams show scintillation counting (cpm, counts per minute) of the cell lysates normalized to cell numbers and to control (*Rtcb*$^{fl/fl}$ or *Rtcb*$^{fl/+}$) mice (n=4, mean and SEM are displayed).
